# Immunogenicity and safety of concomitant administration of a measles, mumps and rubella vaccine (M-M-RvaxPro^®^) and a varicella vaccine (VARIVAX^®^) by intramuscular or subcutaneous routes at separate injection sites: a randomised clinical trial

**DOI:** 10.1186/1741-7015-7-16

**Published:** 2009-04-14

**Authors:** Yves Gillet, Pirmin Habermehl, Stéphane Thomas, Cécile Eymin, Anne Fiquet

**Affiliations:** 1 Urgences Pédiatriques, Hôpital Edouard Herriot, Lyon, France; 2 Facharzt für Kinderheilkunde und Jugendmedizin, Neonatologie, Mainz-Hechtsheim, Germany; 3 Clinical Department, Sanofi Pasteur MSD, Lyon, France

## Abstract

**Background:**

When this trial was initiated, the combined measles, mumps and rubella (MMR) vaccine was licensed for subcutaneous administration in all European countries and for intramuscular administration in some countries, whereas varicella vaccine was licensed only for subcutaneous administration. This study evaluated the intramuscular administration of an MMR vaccine (M-M-RvaxPro^®^) and a varicella vaccine (VARIVAX^®^) compared with the subcutaneous route.

**Methods:**

An open-label randomised trial was performed in France and Germany. Healthy children, aged 12 to18 months, received single injections of M-M-RvaxPro and VARIVAX concomitantly at separate injection sites. Both vaccines were administered either intramuscularly (IM group, *n *= 374) or subcutaneously (SC group, *n *= 378). Immunogenicity was assessed before vaccination and 42 days after vaccination. Injection-site erythema, swelling and pain were recorded from days 0 to 4 after vaccination. Body temperature was monitored daily between 0 and 42 days after vaccination. Other adverse events were recorded up to 42 days after vaccination and serious adverse events until the second study visit.

**Results:**

Antibody response rates at day 42 in the per-protocol set of children initially seronegative to measles, mumps, rubella or varicella were similar between the IM and SC groups for all four antigens. Response rates were 94 to 96% for measles, 98% for both mumps and rubella and 86 to 88% for varicella. For children initially seronegative to varicella, 99% achieved the seroconversion threshold (antibody concentrations of ≥ 1.25 gpELISA units/ml). Erythema and swelling were the most frequently reported injection-site reactions for both vaccines. Most injection-site reactions were of mild intensity or small size (≤ 2.5 cm). There was a trend for lower rates of injection-site erythema and swelling in the IM group. The incidence and nature of systemic adverse events were comparable for the two routes of administration, except varicella-like rashes, which were less frequent in the IM group.

**Conclusion:**

The immunogenicities of M-M-RvaxPro and VARIVAX administered by the intramuscular route were comparable with those following subcutaneous administration, and the tolerability of the two vaccines was comparable regardless of administration route. Integration of both administration routes in the current European indications for the two vaccines will now allow physicians in Europe to choose their preferred administration route in routine clinical practice.

**Trial registration:**

ClinicalTrials.gov NCT00432523

## Background

Although many people consider measles, mumps, rubella and varicella to be 'minor' illnesses, they all carry the risk of serious complications, which may lead to long-term morbidity or death [1-4]. In addition to the distress that these diseases and their complications may cause to affected children and their families, the direct and societal costs of hospitalisation and outpatient visits to manage complications are substantial [5].

The development of vaccines against these four historically common childhood diseases has led to a significant decline in their incidence in countries with routine paediatric vaccination programmes. For example, a two-dose vaccination schedule with the combined measles, mumps and rubella (MMR) vaccine (M-M-R™ II, Merck & Co., Inc.), consisting of the measles virus more attenuated Enders' Edmonston strain, the mumps virus Jeryl Lynn™ (level B) strain and the rubella virus Wistar RA 27/3 strain, has led to elimination of all three diseases in Finland [6] and a greater than 90% reduction in their incidence in Sweden and the USA [7,8]. A live, attenuated varicella vaccine (VARIVAX^®^, Merck & Co., Inc., Oka/Merck strain) was introduced in the USA in 1995. Following its introduction, the age-adjusted annual incidence of varicella in the USA decreased from 2.63 cases per 1000 person-years during 1995 to 0.92 cases per 1000 person-years during 2002, and there was a 75% decrease in incidence among children aged 1 to 4 years between 1992–1996 and 2002 [9]. Furthermore, age-adjusted mortality rates for varicella-associated deaths declined by 66% from 1990–1994 to 1999–2001, with the greatest reduction (92%) seen among children aged 1 to 4 years [10].

Despite the evidence of the reduction in incidence of measles, mumps and rubella that can be achieved by vaccination, and the fact that all member states of the World Health Organization (WHO) Europe Region have adopted a two-dose measles vaccination schedule, the coverage rates for MMR vaccination in Europe, particularly with respect to the second dose, are still too low to achieve the WHO objective of eliminating all three diseases in Europe by 2010 [11-13]. Furthermore, although the USA has demonstrated the benefits of universal vaccination of children aged 12 to 18 months against varicella [14], Germany [15] and some regions of Italy and Spain [16] are currently the only parts of Europe to recommend universal childhood vaccination against varicella. Other European countries have yet to fully evaluate and adopt varicella vaccination programmes [17,18].

Worldwide, over 500 million doses of M-M-R II have been distributed, and it has been shown to be generally well tolerated, immunogenic and efficacious [19,20]. M-M-R II has been proven to be effective in preventing the development of measles (91% to 100% efficacy) [21,22], mumps (75% to 96% efficacy) [23,24] and rubella (93% to 100% efficacy) [25,26].

M-M-RvaxPro^®^, a new version of M-M-R II that contains the same components, obtained its marketing authorisation in Europe in May 2006. The only difference between M-M-R II and M-M-RvaxPro resides in the replacement of human serum albumin in M-M-R II with recombinant human albumin during the manufacturing of measles, mumps and rubella viral bulks. The immunogenicity and safety profiles of M-M-RvaxPro have been shown to be comparable with those of M-M-R II [27].

VARIVAX has also been shown to be immunogenic, effective and well tolerated. The results from a study on 1164 healthy children concluded that an antibody concentration of ≥ 5 glycoprotein (gp) enzyme-linked immunosorbent assay (ELISA) units/ml could be regarded as an approximate correlate of protection for individual vaccinees [28]. Seroconversion rates (increase above the threshold of ≥ 1.25 gpELISA units/ml) after administration of a single dose of VARIVAX range from 94% to 96% [29,30]. In clinical practice, the efficacy of VARIVAX is approximately 85% against all forms of varicella and about 97% against moderately severe to severe forms [31-33].

The opportunity to administer vaccines concomitantly can improve compliance with paediatric vaccination regimens and enhance vaccine coverage [34]. It has been demonstrated previously that M-M-R II and VARIVAX, administered subcutaneously at two separate injection sites during the same visit to children aged between 12 months and 6 years, are generally well tolerated and immunogenic, with protection against varicella maintained for up to 6 years [35].

In Europe, recommendations and preferred practices vary with regard to the route of vaccination. At the start of the study, the MMR vaccine from Merck was licensed for subcutaneous (SC) administration in all European countries, and in some countries for both SC and intramuscular (IM) administration, whereas the varicella vaccine was licensed only for SC administration. It is generally accepted, however, that many physicians prefer to administer vaccines intramuscularly. This study was thus performed to compare the immunogenicity and safety of M-M-RvaxPro and VARIVAX when both are administered concomitantly at separate injection sites by either the SC or the IM route.

## Methods

### Study design and conduct

This open-label, randomised trial was performed in France (39 centres) and Germany (33 centres) between January and September 2005. The study was performed in accordance with the principles of the Declaration of Helsinki and of Good Clinical Practice and was registered on the European Communities trial registry . Approval was obtained from the relevant competent authorities and ethics committees (CCPPRB (Comité Consultatif pour la Protection des Personnes dans la Recherche Biomédicale) of Lyon A, France and independent ethics committee of Rheinland Pfalz, Mainz, Germany) before the trial began. Written informed consent was obtained from parents or legal guardians of all children before enrolment in the study.

### Study population and vaccines

Healthy children of either sex, between 12 and 18 months of age, were eligible for inclusion in the study. Participants were required to have no history of vaccination for measles, mumps, rubella or varicella, and no suspected clinical history or exposure in the past 30 days to these diseases. They should also have had no history of febrile illness (rectal temperature ≥ 38°C) within the 3 days before enrolment, and to have no known sensitivity or allergy to any component of the study vaccines. Children who had received any vaccine in the 30 days before enrolment were not eligible for inclusion. Children with immune impairment or humoral and/or cellular deficiency, neoplastic disease or depressed immunity, including that resulting from long-term (≥ 14 days) corticosteroid administration at high doses, were also not eligible.

Group allocation was performed using balanced permuted blocks of randomisation of size 4. The randomisation was stratified by centre and generated by a non-study statistician from the clinical research organisation S-Clinica. The investigators called an interactive voice response system (to avoid subject selection bias) to determine which administration route (IM or SC) was to be used. Infants received single concomitant doses of M-M-RvaxPro and VARIVAX, either both by IM or both by SC, at separate injection sites in the upper arm. The use of anaesthetic preparations at the injection sites was not permitted. To decrease the chances of the investigators being biased by remembering the route of administration for the safety assessment at the second study visit (42 to 56 days after vaccination), they were instructed to record the route of administration in the vaccination log and in the relevant section of the case report form and then to seal these documents in an envelope that could not be opened until after the second visit.

M-M-RvaxPro is a lyophilised live virus vaccine manufactured with recombinant human albumin for vaccination against measles, mumps and rubella. The 50% cell-culture-infectious dose was not less than 1 × 10^3 ^for measles (derived from attenuated Enders' Edmonston strain) and rubella (Wistar RA 27/3 strain), and not less than 12.5 × 10^3 ^for mumps (Jeryl Lynn [Level B] strain). VARIVAX is a lyophilised preparation of the live Oka strain varicella virus, with a potency of not less than 1350 plaque-forming units. Both vaccines were stored at 2°C to 8°C and reconstituted with sterile water for injections immediately before use.

### Subcutaneous and intramuscular vaccination techniques

The needles used to reconstitute the vaccines were replaced with fresh 25-gauge 16 mm-long needles before injection. For IM injections, the investigators were advised to use their thumb and index finger to stretch the skin flat over the deltoid muscle to insert the needle perpendicular to the skin and then to inject the vaccine. For the SC injections, the investigators were advised to hold the skin and subcutaneous tissue in the deltoid area between their thumb and fingers thus producing a skin fold, to insert the needle into the fold at about 45° and then inject the vaccine while taking care to deliver it only in the SC tissue.

### Objectives

The primary objectives of the study were to assess if, in children aged 12 to 18 months at 42 days following vaccination:

• a single dose of M-M-RvaxPro administered by the IM route concomitantly with VARIVAX by the same route at separate injection sites;

• and/or, a single dose of VARIVAX administered by the IM route concomitantly with M-M-RvaxPro by the same route at separate injection sites

were, in terms of response rates, non-inferior compared with the same vaccine administered by the SC route. Success was defined in this study as either one or both primary objectives being reached, therefore the type 1 error rate was adjusted using the method described in the statistical methods below.

The secondary objectives of the study were to determine the antibody concentrations to measles, mumps, rubella and varicella at 42 days after concomitant vaccination and to evaluate the safety profiles of the two vaccines and routes of administration.

### Assessments

For assessment of immunogenicity, a 5 ml blood sample was taken before vaccination and at the second study visit (days 42 to 56 days after vaccination). The concentrations of antibodies were determined by Merck Research Laboratories (West Point, Pennsylvania, USA) on the serum samples using ELISA for measles, mumps and rubella and a gpELISA for varicella [36-38].

The primary immunogenicity criteria were the response rates to measles, mumps and rubella and varicella defined as the percentage of subjects who had been seronegative before vaccination and who developed antibody concentrations of ≥ 255 mIU/ml for measles, ≥ 10 ELISA Ab units for mumps, ≥ 10IU/ml for rubella and ≥ 5gpELISA units/ml for varicella 42 days following vaccination. In addition, we also ascertained the seroconversion rate for varicella defined as the percentage of subjects who were seronegative before vaccination and who developed post-vaccination antibody concentrations of ≥ 1.25gpELISA units/ml. The secondary immunogenicity criteria were the post-vaccination geometric mean concentrations (GMCs) of antibodies to measles, mumps, rubella and varicella.

Parents or legal guardians were asked to record all injection-site reactions and any systemic adverse events in a diary card from day 0 (day of vaccination) until day 42 post vaccination. Solicited injection-site reactions (erythema, swelling and pain) were recorded on days 0 to 4. Other injection-site reactions (spontaneously reported), daily axillary temperature, measles-, rubella- or varicella-like rashes, and mumps-like symptoms, and any other systemic adverse events were recorded from days 0 to 42. Serious adverse events were recorded from day 0 until the final study visit. The parents or legal guardians were asked to notify the investigator if their child developed a measles-, rubella- or varicella-like rash or mumps-like illness, or if they experienced a serious adverse event. They were also instructed to measure the rectal temperature if their child's axillary temperature was ≥ 37.1°C.

### Statistical analyses

The primary hypotheses were that the response rates defined for each antigen as primary criteria for children in the IM group would not be inferior to those for children in the SC group for one or both vaccines. The sample size calculation was based on these primary hypotheses. The immunogenicity analysis was performed on a per-protocol set of subjects (PPS) that included all randomised children, except those with a protocol violation that might have interfered with the immunogenicity evaluation – main analysis and on a full-analysis set (FAS) that included all randomised children who received at least one study vaccine and for whom a post-vaccination immunogenicity evaluation was available. Only children who were seronegative at baseline were included in the PPS analysis for the corresponding antigen (antigen-specific PPS) whereas all subjects were included in the FAS, regardless of their initial serostatus. The statistical analysis for the primary endpoint was based on two-sided confidence intervals (CI) around the difference in response rates [IM – SC] for each vaccine antigen. The analysis for the demonstration of the non-inferiority of response rates for the IM group compared with the SC group was based on the method proposed by Miettinen and Nurminen [39] with stratification by region (that is, pooled data from centres based on geographic location). In addition we performed non-stratified analyses to verify the robustness of the primary results.

The non-inferiority criterion for each antigen was achieved if the lower limit of the CI was above – 10%. To adjust for multiple statistical testing we used the method proposed by Hochberg [40], which leads to different CI limits, depending on the results of the study (95% if non-inferiority was demonstrated for both vaccines and 97.5% in case of failure of one vaccine).

Descriptive analyses were performed for measles, mumps, rubella and varicella GMCs (with 95% CI). The safety analysis was performed on all children who received at least one of the study vaccines and for whom safety follow-up data were available. The analysis was performed according to the route actually used for vaccination. Children with vaccination errors were excluded from the safety analysis; however, their safety data were carefully reviewed.

All statistical analyses were performed using SAS^® ^software version 8.2 (SAS Institute Inc., Cary, North Carolina, USA).

## Results

### Subjects

In total, 752 children were randomised and received M-M-RvaxPro and VARIVAX either by the intramuscular (*n *= 374) or subcutaneous (*n *= 378) route.

Overall, the mean age at vaccination was 13.7 ± 1.7 months, and there were slightly more boys (416; 55.3%) than girls (336; 44.7%) included in the study. Children in the two vaccination groups were comparable with respect to age at vaccination, gender, weight and height (Table 1). The two groups were also comparable in the percentages of children who were seronegative at baseline for measles, mumps, rubella and varicella (Table 1).

**Table 1:** Demographic and baseline data in the randomised set.

	**IM Group** *** N *= 374**	**SC Group** *** N *= 378**
Age at vaccination, months ± SD	13.8 ± 1.7	13.7 ± 1.6

Male, *n *(%)	206 (55.1)	210 (55.6)

Weight, kg ± SD	10.1 ± 1.2	10.2 ± 1.3

Height, cm ± SD	77.2 ± 3.6	77.4 ± 3.4

Seronegative, n (%)^1^		
Measles (<255 mIU/ml)	364 (97.3)	371 (98.1)
Mumps (<10 ELISA Ab units/ml)	364 (97.3)	370 (97.9)
Rubella (<10 IU/ml)	335 (89.6)	324 (85.7)
Varicella (<1.25 gpELISA units/ml)	352 (94.1)	353 (93.4)

(IM group, both vaccines administered by the intramuscular route; SC group, both vaccines administered by the subcutaneous route).

^1 ^It is not possible to calculate the seropositive rates directly from these data because of the missing values; overall 5 (0.7%) subjects were seropositive at baseline for measles, 11 (1.5%) for mumps, 81 (10.8%) for rubella and 37 (4.9%) for varicella

### Immunogenicity

The composition of the antigen-specific PPS is detailed in Table 2. In each antigen-specific PPS, the antibody response rates at 42 days post vaccination for children initially seronegative to measles, mumps, rubella or varicella were non-inferior in the IM compared with the SC groups (Table 3). In both groups, response rates were 94% to 96% for measles, and 98% for mumps and rubella. For varicella, 86% of children in the SC group and 88% in the IM group had antibody concentrations ≥ 5gpELISA units/ml, while 98.5% (96.6, 99.5) and 99.4% (97.9, 99.9) in the IM and SC groups, respectively, had concentrations ≥ 1.25gpELISA units/ml. The lowest lower limit for the 95% CIs of the response rates for the four antigens was -5.28% (for measles). The primary hypothesis that the intramuscular route of administration is not inferior to the subcutaneous route was therefore met for all antigens and thus for both vaccines. The immunogenicity results for the FAS were comparable with those for each PPS, and results from the sensitivity analyses showed they were robust (data not shown).

**Table 2:** Composition of the antigen-specific per protocol sets (PPSs)

	IM Group				SC Group			
	Measles	Mumps	Rubella	Varicella	Measles	Mumps	Rubella	Varicella

Randomised	374	374	374	374	378	378	378	378

Vaccinated	374	374	374	374	378	378	378	378

Analysed in PPS	349	349	321	336	363	363	318	345

Not included in PPS^1^	25	25	53	38	15	15	60	33

- Seropositive at baseline	3	7	32	18	2	4	49	19

- Protocol deviations^2^	23	19	23	20	13	11	13	14^3^

(IM group, both vaccines administered by the intramuscular route; SC group, both vaccines administered by the subcutaneous route).

^1 ^An infant could have more than one reason for not being included in the PPS, and some deviations do not apply to all valences; for example, data for one valence could be missing because there was not enough serum to perform the assay.

^2 ^Protocol deviations included: previous vaccination for measles, mumps rubella or varicella; received non-study vaccine close to inclusion; route of vaccination different from that allocated by randomisation; initial serostatus unknown (first blood sample not taken or missing data), post-vaccination data missing (blood sample not taken or missing data); blood sample not taken at the right time; received only vaccine diluent.

^3 ^Includes one subject exposed to varicella during follow-up.

**Table 3:** Response rates to measles, mumps, rubella and varicella at 42 days post vaccination for children initially seronegative to measles, mumps, rubella or varicella in the antigen-specific per-protocol sets

	**IM Group** ***N *= 374^1^**		**SC Group** ***N *= 378^1^**		**Difference (%)^3^** **IM group – SC group** **[95% CI]**
	
	***n*^2^**	**Response rate** ***n *(%)^4^** **[95% CI]**	***n*^2^**	**Response rate** ***n *(%)^4^** **[95% CI]**	
Measles (≥ 255 mIU/Ml)	349	329 (94.3) [91.3; 96.5]	363	349 (96.1) [93.6; 97.9]	-1.89 [-5.28; 1.29]

Mumps (≥ 10 ELISA Ab units/ml)	349	341 (97.7) [95.5; 99.0]	363	356 (98.1) [96.1; 99.2]	-0.33 [-2.67; 2.00]

Rubella (≥ 10 IU/ml)	321	315 (98.1) [96.0; 99.3]	318	312 (98.1) [95.9; 99.3]	-0.02 [-2.42; 2.43]

Varicella (≥ 5 gpELISA units/ml)	336	297 (88.4) [84.5; 91.6]	345	295 (85.5) [81.3; 89.0]	2.93 [-2.18; 8.06]

(IM group, both vaccines administered by the intramuscular route; SC group, both vaccines administered by the subcutaneous route; ELISA, enzyme-linked immunosorbent assay; Ab, antibody; gpELISA, glycoprotein antigen-based enzyme-linked immunosorbent assay.)

^1^Number of vaccinated subjects

^2^Number of subjects initially seronegative to measles (<255 mIU/ml), mumps (<10 ELISA Ab units/ml), rubella (<10 IU/ml) or varicella (<1.25 gpELISA units/ml) contributing to each per-protocol set

^3^Stratified by region

^4^Number and percentage of subjects

**Figure 1:**
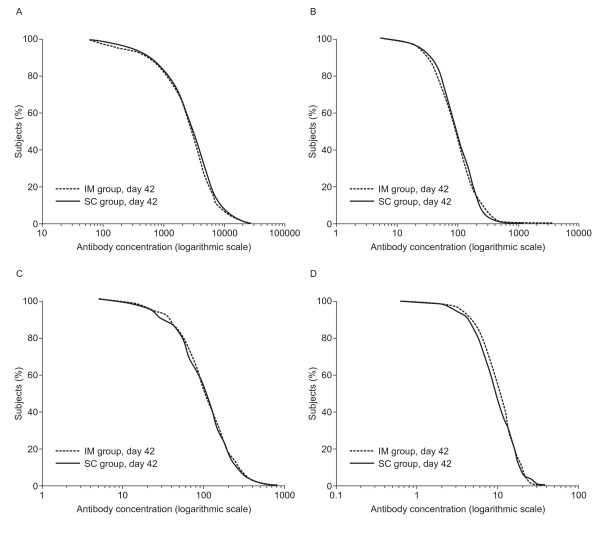
**Reverse cumulative distribution curve of antibody concentrations for (a) measles, (b) mumps, (c) rubella and (d) varicella at 42 days post-vaccination for children initially seronegative to measles, mumps, rubella or varicella in the antigen-specific per-protocol sets**. (IM group, both vaccines administered by the intramuscular route; SC group, both vaccines administered by the subcutaneous route).

In each antigen-specific PPS, the GMCs at day 42 for antibodies against measles, mumps, rubella and varicella in children initially seronegative to the corresponding antigen were comparable between the IM and SC groups (Table 4). The reverse cumulative distribution curves, for each valence, confirm the similarity of the response rates between groups (Figure 1).

**Table 4:** Geometric mean concentrations (GMC) of measles, mumps, rubella and varicella antibodies at 42 days post-vaccination for children initially seronegative to measles, mumps, rubella or varicella in the antigen-specific per-protocol sets

	**IM Group** ***N *= 374^1^**			**SC Group** ***N *= 378^1^**		
	
	***n*^2^**	**GMC**	**[95% CI]**	***n*^2^**	**GMC**	**[95% CI]**
Measles (mIU/ml)	349	2396.4	[2117.7; 2711.8]	363	2560.6	[2278.5; 2877.7]

Mumps (ELISA Ab units/ml)	349	86.4	[78.7; 95.0]	363	89.8	[82.6; 97.6]

Rubella (IU/ml)	321	97.2	[88.6; 106.7]	318	94.4	[85.7; 104.0]

Varicella (gpELISA units/ml)	333	9.8	[9.2; 10.5]	345	9.2	[8.6; 9.8]

(IM group, both vaccines administered by the intramuscular route; SC group, both vaccines administered by the subcutaneous route; ELISA, enzyme-linked immunosorbent assay; Ab, antibody, gpELISA, glycoprotein antigen-based enzyme-linked immunosorbent assay.)

^1^Number of vaccinated subjects

^2^Number of subjects initially seronegative to measles, mumps, rubella or varicella contributing to each per-protocol set

### Safety

One child in the IM group was withdrawn from the study for personal reasons and one in the SC group did not attend the second (last) study visit; however, safety data for both children were collected by telephone at the time of visit 2. No child was withdrawn from the study because of an adverse event.

One child who was randomised to the SC group but received both vaccines by the intramuscular route was included in the IM group for the safety analysis and two children (one from each group) were excluded from the safety analysis due to vaccination errors; one child received only the diluent of M-M-RvaxPro and the other child received M-M-RvaxPro by deep subcutaneous injection. The safety analysis was thus performed on 374 children in the IM group and 376 children in the SC group.

A total of 313 (83.7%) children in the IM group and 325 (86.4%) children in the SC group had at least one adverse event (injection-site reaction or systemic adverse event) within 42 days following vaccination. In both groups, approximately half of the children experienced at least one adverse event (injection-site reaction or systemic adverse event) related to each vaccine: 50.8% in the IM group and 53.7% in the SC group had an adverse event assessed by the investigators as being related to vaccination with M-M-RvaxPro. The corresponding rates for VARIVAX were 46.3% and 55.9%, respectively.

For both vaccines and both administration routes, most injection-site reactions were of mild intensity or small size (≤ 2.5cm). Although not formally tested, there was a trend for lower rates for solicited injection-site reactions on days 0 to 4 in the IM group compared with the SC group (Table 5). Most solicited injection-site reactions occurred on the day of vaccination and resolved within 1 day. The same trend for lower rates of erythema and swelling was observed on days 5 to 42 for both vaccines in the IM group (M-M-RvaxPro: erythema 0.3% and swelling 0% in the IM group compared with erythema 4.3% and swelling 1.6% in the SC group; VARIVAX: erythema 4.8% and swelling 1.6% in the IM group compared with erythema 12.2% and swelling 5.3% in the SC group).

**Table 5:** Number and percentage of children with a solicited injection-site reaction on days 0 to 4 in the safety set

	**M-M-RvaxPro**		**VARIVAX**	
	
	**IM group** ***N *= 374^1^**	**SC group** ***N *= 376^1^**	**IM group** ***N *= 374^1^**	**SC group** ***N *= 376^1^**
	**Number of subjects (%)^2^**			

Solicited injection-site reaction	58 (15.5)	81 (21.5)	57 (15.2)	85 (22.6)

Erythema	39 (10.4)	61 (16.2)	33 (8.8)	63 (16.8)

Pain	26 (7.0)	27 (7.2)	26 (7.0)	32 (8.5)

Swelling	7 (1.9)	20 (5.3)	12 (3.2)	18 (4.8)

(IM group, both vaccines administered by the intramuscular route; SC group, both vaccines administered by the subcutaneous route.)

^1^Number of vaccinated subjects according to the protocol and the actual route of administration

^2 ^Number (and percentage) of subjects presenting the reaction at least once

The incidences of measles- and rubella-like rashes and of mumps-like symptoms were similar in both groups (Table 6). Generalised varicella-like rashes were reported less frequently in the IM group compared with the SC group. The proportions of children who experienced a maximal rectal temperature of 39.4°C or above between days 0 and 42 were comparable for the two groups: 20.9% (78/373) in the IM group and 22.5% (84/374) in the SC group. Elevated rectal temperatures occurred mainly between days 5 and 12 in both groups.

**Table 6:** Number and percentage of children with a systemic adverse event on days 0 and 42 in the safety set

	**IM group** ***N *= 374^1^**	**SC group** ***N *= 376^1^**
	
	**Number of subjects (%)^2^**	
Systemic adverse event	295 (78.9)	295 (78.5)

Vaccine-related systemic adverse event	156 (41.7)	156 (41.5)
Related to M-M-RvaxPro	153 (40.9)	149 (39.6)
Related to VARIVAX	121 (32.4)	125 (33.2)

Measles-like rash^3^	11 (2.9)	10 (2.7)

Mumps-like illness	0	1 (0.3)

Rubella-like rash^3^	10 (2.7)	10 (2.7)

Varicella-like rash^3^	2 (0.5)	12 (3.2)

(IM group, both vaccines administered by the intramuscular route; SC group, both vaccines administered by the subcutaneous route)

^1^Number of vaccinated subjects according to the protocol and the actual route of administration

^2 ^Number (and percentage) of subjects presenting the event at least once

^3^Non-injection site rash

Five children experienced a serious adverse event (one in the IM group and four in the SC group). One of these events – otitis media of moderate intensity reported for a child at day 5 post vaccination in the SC group – was assessed by the investigator as possibly related to both vaccines.

## Discussion

This European multicentre study has demonstrated that concomitant IM administration of M-M-RvaxPro and VARIVAX elicits an immune response that is comparable with that following SC administration with respect to response rates to measles, mumps, rubella and varicella at 42 days post vaccination. Comparable antibody concentrations were observed for each vaccine, whether given by the IM or the SC route. Both vaccines were generally well-tolerated and the safety profile for both injection routes was also globally comparable.

Immunogenicity results for the FAS were comparable with those for each antigen-specific PPS, indicating that both vaccines are likely to be effective when administered by either route in routine clinical practice. The response rates to M-M-RvaxPro in this study were comparable with those reported previously, and the levels of immunogenicity observed were consistent with the antibody concentrations known to provide protection against measles, mumps and rubella. Similarly, response rates to VARIVAX were within the expected range, regardless of the route of administration, and were consistent with those reported previously from a comparative study of the IM versus SC administration of the Oka/Merck vaccine in healthy children in the USA [41].

Although the trial was limited by being open label, details of the administration route were sealed in an envelope after the vaccines had been administered to reduce possible investigator bias during the second study visit. In addition, the randomisation was performed using an interactive voice response system, thus minimising any subject selection bias. Consistent with other vaccine trials, the reporting of injection-site reactions and systemic adverse events, other than erythema, swelling and pain from days 0 to 4 post-injection, was spontaneous (that is, not solicited). It is notable that the present study had an excellent completion rate. All randomised children were vaccinated and only two could not be included in the safety analysis.

The safety profile of concomitant M-M-RvaxPro and VARIVAX administration in this study was consistent with that expected from previous studies of the two vaccines, with the most common adverse events being injection-site reactions [41,42]. The overall tolerability profile of the two vaccines was comparable for the IM and SC routes of administration. As expected, however, there was a trend for lower rates of injection-site reactions following IM administration. Generalised varicella-like rashes were also observed less frequently when VARIVAX was administered by the IM route, as has been reported previously [41].

Use of the IM route for paediatric vaccinations is common practice in Europe. As recommendations and physician preferences vary, there is a need for greater flexibility in routes of vaccine administration. The present study has shown that the immunogenicities of M-M-RvaxPro and VARIVAX administered concomitantly by the IM route are comparable with the immunogenicities of both vaccines administered by the SC route. The better tolerability of both vaccines when administered by the IM route may help to improve compliance with vaccination schedules. This is particularly important for ensuring that children receive a second MMR dose (as recommended in most countries), because coverage is currently sub-optimal [13].

These data have been used to apply successfully for modification to the licence for both M-M-RvaxPro and VARIVAX, so that both SC and IM administration is now possible.

## Conclusion

The immunogenicity of M-M-RvaxPro and VARIVAX administered by the IM route was comparable with that for the two vaccines administered by the SC route. In addition, the globally comparable tolerability of both vaccines, regardless of the administration route, may help to improve compliance with vaccination schedules as flexibility in the route of administration is now possible for both vaccines. This allows physicians to exercise their personal preference in routine practice and would be consistent with common practice in many parts of Europe. This should help to increase vaccine coverage, leading to improved protection and reduced incidence of measles, mumps, rubella and varicella in Europe.

## Competing interests

YG and PH have declared that they have no competing interests. ST, CE and AF are employed by Sanofi Pasteur MSD, which markets the study vaccines.

## Authors' contributions

YG participated in the trial design, data analysis and critical review of the manuscript. PH participated in data collection, and critical review of the manuscript. ST was responsible for trial design, analysis and interpretation of data, and critical review of the manuscript. CE participated in data collection and study coordination. AF was responsible for trial design, protocol writing, overall conduct of the study, analysis and interpretation of data, and critical review of the manuscript. All authors read and approved the final manuscript.

## Pre-publication history

The pre-publication history for this paper can be accessed here:


